# Factors associated with sex in the context of methamphetamine use in different sexual venues among HIV-positive men who have sex with men

**DOI:** 10.1186/1471-2458-10-178

**Published:** 2010-04-01

**Authors:** Shirley J Semple, Steffanie A Strathdee, Jim Zians, Thomas L Patterson

**Affiliations:** 1 Department of Psychiatry (MC 0680), University of California - San Diego, 9500 Gilman Drive, La Jolla, California 92093-0680, USA; 2 Division of Global Public Health, Department of Medicine (MC 0507), University of California - San Diego, 9500 Gilman Drive, La Jolla, California 92093-0507, USA

## Abstract

**Background:**

Harm reduction has focused primarily on reduction of high-risk substance using behaviors rather than reductions in high-risk sexual behaviors. Furthermore, most studies focus on individual behavior change, with less attention paid to the social and environmental context. This paper promotes understanding of the interplay between the individual and the social context by examining the psychosocial and behavioral characteristics of 321 methamphetamine-using HIV-positive men who have sex with men (MSM) in San Diego, CA based on the locations or venues of their sexual activities when "high" on methamphetamine.

**Methods:**

Participants in a safer-sex intervention study underwent a baseline assessment that queried demographic and psychosocial characteristics as well as drug use and sexual risk behaviors. For purposes of analysis, respondents were classified according to their preference of sexual venue: private (e.g., home), commercial (e.g., bathhouse), or public (e.g., public park or restroom).

**Results:**

The commercial venue group was younger, better educated, more likely to identify as gay, and significantly more likely to have used "club drugs" as compared to the other two groups. Men in the commercial- and public-venue groups reported more high-risk sex compared to the private-venue group. The public-venue group reported heavier drug and alcohol use, had significantly higher Beck depression scores, reported more experiences of stigma, and scored higher on a measure of sexual compulsivity than did the other two groups.

**Conclusion:**

In an effort to reduce HIV/STI risk-behaviors, future studies should investigate the feasibility of modifying personal, psychosocial and structural factors associated with the use of risky sexual venues where HIV-positive methamphetamine users engage in sexual activity when "high" on methamphetamine.

**Trial registration:**

ClinicalTrials.gov NCT00432926

## Background

Methamphetamine use among men who have sex with men (MSM) has been associated with high rates of sexually transmitted infections, low rates of condom use, high rates of unprotected anal sex, prolonged sexual activity, multiple partners, and casual partners [1-4]. In recent years, researchers have studied the complex array of factors that affect HIV risk behaviors in this population. We reported findings from a behavioral intervention among HIV-positive, methamphetamine-using MSM that demonstrated that it was possible to reduce high-risk sexual behaviors among this population, even in the context of ongoing methamphetamine use [5]. To date, however, little research has focused on sexual venues and the risk behaviors of HIV-positive, methamphetamine-using MSM. Enhanced understanding of the interplay between individual behavior and social context could lead to more effective behavioral interventions and sexual harm reduction initiatives [6].

Several studies have revealed that sexual risk behaviors of HIV-negative MSM vary according to sexual venue. Coates and colleagues reported that MSM who met their partners in sex clubs and bathhouses had significantly more unprotected anal intercourse and used more drugs and alcohol than did men who did not attend these venues [7]. In two studies involving Latino MSM, Diaz and colleagues found an association between public sex environments and higher levels of unprotected anal sex and illicit drug use [8,9]. Ekstrand and colleagues found an association between high-risk sexual behaviors and attendance at commercial sex venues [10]. In a large-scale study of MSM, Binson and colleagues reported significant associations between demographic characteristics, attendance at sexual venues, and risky sexual behaviors [11].

Parsons and Halkitis compared the sexual risk behaviors, drug use behaviors, and psychosocial characteristics of HIV-positive MSM who attended commercial and public sex environments with those who did not [12]. MSM who frequented non-commercial, public sex environments were more "sexually compulsive" and engaged in more sexual risk-behavior as compared to men who did not. MSM who frequented commercial sex environments reported higher levels of depression and were more likely to use amphetamines, ecstasy, hallucinogens, and poppers. To date, the Parsons and Halkitis paper appears to be the only published study of sexual venues among HIV-positive MSM. Given the close relationships between high-risk sex, methamphetamine use, and sexual venues, we designed this study to help inform the development of behavioral interventions and harm reduction initiatives in social-sexual environments. Three research questions were addressed: 1) What type of sexual venues are most frequently used by HIV-positive methamphetamine-using MSM when they have sex and are "high" on methamphetamine?; (2) What types and levels of sexual- and drug-risk behavior are associated with different types of venues where sexual activity and methamphetamine use are combined?; and (3) Are demographic characteristics, substance-use variables, and psychosocial factors associated with specific patterns of attendance at venues where sexual activity is combined with methamphetamine use?

## Methods

### Sample selection

The sample consisted of 321 HIV-positive, methamphetamine-using MSM who participated in the EDGE research project at the University of California, San Diego between November 2000 and November 2004. The eight-session protocol used principles of social-cognitive theory, theory of reasoned action, and motivational interviewing to reduce the sexual risk practices of HIV-positive, methamphetamine-using MSM [13-15]. Eligibility criteria were: (a) self-identified as MSM; (b) HIV-positive for at least two months; (c) used methamphetamine at least twice in the past two months and at least once in the past 30 days; and (d) reported having unprotected anal or oral sex with at least one HIV-negative or serostatus-unknown male partner during the previous two months. Recruitment strategies included community outreach in gay-identified venues, posters, advertisements, referrals from service providers, and informal referrals. The study was approved by the Human Research Protections Program at the University of California, San Diego (UCSD IRB #050417), and all participants provided informed consent.

### Procedures

Data were taken from the baseline interview, which covered a range of topics including methamphetamine use patterns, use of alcohol and other substances, sexual risk practices with HIV-negative and serostatus-unknown partners, HIV-related attitudes, sexual communication skills, disclosure behaviors, and social-cognitive factors.

### Measures

Measures of methamphetamine included amount (number of grams consumed in the past 30 days), frequency (number of days used in the last 30 days), binge use, and injection use (yes/no in last two months). Alcohol use was measured using three items that inquired about frequency of use, frequency of intoxication, and number of drinks consumed on a typical day.

Sexual risk behavior was defined as unprotected anal, oral, or vaginal sex with an opposite- or same-sex partner during the previous two months. Partner types were steady, casual, and anonymous, and for each type, participants were asked how many times during the past two months they engaged in protected or unprotected sex of the following kinds: receptive anal sex; insertive anal sex; receptive (passive) oral sex; insertive (i.e., giving) oral sex; and insertive vaginal sex. Number of sexually transmitted infections in the previous two months was determined by self-report.

Participants were presented with a list of twelve venues compiled in qualitative work [16] and were asked: "During the past two months, when you were high on methamphetamine, did you have sex in any of the following locations?" An open-ended response category was also included. Thematic analysis revealed three broad categories of participants according to sexual venues used during the past two months. The first category, "private venues only," comprised participants (N = 100) who had had sex exclusively in either their own homes or in a sex partner's home. The second category, "at least one commercial venue, but no public venues," included individuals (N = 114) who had had sex in a commercial location (e.g., bathhouse, sex club, adult bookstore, gay theatre, bar, dance club, after-hours club) but reported no sex in a public location. Most participants in this second category had also had sex in a private location. The third category, "at least one public venue," consisted of individuals (N = 107) who reported having had sex in a public location (e.g., park, public restroom, street corner). The majority of individuals in this category had also had sex in a private location and in a commercial location. Open-ended response categories were coded by three researchers. For example, sexual activity at a family member's home or in a motel room was coded "private venues." Inter-rater reliability between the three coders was high (kappa = .99).

Depressive symptoms were measured by the Beck Depression Inventory (BDI) [17,18], and sexual compulsivity was measured using the scale of Kalichman and colleagues [19]. Mean scores were calculated for two dimensions of social stigma: an 8-item "expectations of rejection" scale and a 6-item "experiences of stigma" scale [20].

### Data analysis

The following variables had skewed distributions that required a log10 transformation: total number of unprotected sex acts; number of unprotected anal sex acts; number of unprotected oral sex acts; number grams of methamphetamine used; and number of months HIV-positive. Our three sexual-venue groups were compared in terms of demographic characteristics, substance use, HIV risk behaviors, and psychosocial factors. One-way ANOVA was used to compare group means. Pairwise multiple comparisons (p < .05) were used to determine group mean differences. Cross-tabulations were conducted to determine group differences for all categorical variables. Pearson chi-square and the likelihood ratio Chi-square were used to determine statistically significant differences.

## Results

### Demographic characteristics

Of the 321 participants, mean age was 37.1 years (range: 20 to 61). Fifty-eight percent were Caucasian, 13% were Latino, 21% were African American, and 8% were "other." Eighty-two percent self-identified as gay or homosexual. Over half (60%) had at least some college education. The majority lived alone or with other adults in a non-sexual relationship (32% and 31%, respectively). Seventy-three percent were unemployed, and 78% reported an annual income of less than or equal to $19,999. Participants had been HIV-positive for an average of 84 months. Rates of unprotected sex were as follows: oral and anal sex (69.5%), oral sex only (30.5%).

As shown in Table 1, men in the commercial-but-no-public-venues group were significantly younger, better educated, and more likely to self-identify as gay or homosexual compared to men in the other two groups. Moreover, men in the public-venues group were significantly more likely to report an "other" living arrangement (e.g., homelessness, community shelter) compared to men in the private-venues-only group. The three groups did not differ significantly on ethnicity, employment status, income, or number of months HIV-positive.

**Table 1:** Comparison of sexual venue groups on substance use, sexual risk behaviors, and psychosocial factors (N = 321)

Variable		Private venues only (N = 100)	Commercial but no public venues (N = 114)	Public venues (N = 107)	Test statistic	*df*	*p*-value
**Demographic characteristics**							
Age in years, mean (SD) (range)		38.0 (8.0) (20-61)	35.4 (7.0) (21-55)	38.2 (6.7) (23-56)	F = 5.5	2,318	< .01
*Sexual orientation*	Gay or homosexual	74.0%	90.6%	74.8%	χ^2 ^= 14.9	4	< .01
	Bisexual	24.0%	9.4%	25.2%			
	Not sure	2.1%	0.0%	0.0%			
*Education*	Less than high school	17.0%	7.0%	17.8%	χ^2 ^= 22.6	6	< .001
	High school or equivalent	25.0%	17.5%	36.4%			
	Some college	35.0%	43.9%	30.8%			
	College or advanced degree	23.0%	31.6%	15.0%			
*Living arrangement*	Living with spouse or steady	24.0%	14.0%	10.3%	χ^2 ^= 15.4	6	< .05
	Living with other adult(s)	23.0%	36.8%	32.7%			
	Living alone	37.0%	31.6%	29.0%			
	Other	16.0%	17.5%	28.0%			
**Substance use**							
Number grams of meth used in past 30 days, mean (SD)		4.4 (7.2)	4.2 (10.4)	7.3 (13.9)	F = 5.1	2,298	< .01
Injected meth in past 2 months		40.0%	30.7%	52.3%	χ^2 ^= 10.7	2	< .01
Used cocaine in past two months		26.0%	22.8%	40.2%	χ^2 ^= 8.9	2	< .01
Used crack in past two months		14.0%	12.3%	33.0%	χ^2 ^= 17.9	2	< .001
Used heroin in past two months		5.0%	2.6%	12.1%	χ^2 ^= 8.7	2	< .01
Used poppers in past two months		45.9%	72.8%	57.5%	χ^2 ^= 16.0	2	< .001
Used Special K in past two months		10.0%	28.1%	13.1%	χ^2 ^= 14.2	2	< .001
Used GHB in past two months		20.0%	38.6%	15.9%	χ^2 ^= 17.2	2	< .001
Number of illicit drugs used, mean (SD)		2.7 (1.8)	3.6 (2.0)	3.5 (2.5)	F = 5.4	2,318	< .01
Number of alcoholic drinks in a typical day, mean (SD)		3.2 (4.3)	2.8 (3.5)	4.5 (5.6)	F = 4.2	2,315	< .05
Became drunk from alcohol in past two months		33.3%	46.0%	57.0%	χ^2 ^= 11.6	2	< .01
**Sexual risk behavior**							
Total unprotected sex acts, mean (SD)		30.1 (48.0)	33.7 (40.7)	42.5 (55.5)	F = 3.6	2,305	< .05
Unprotected anal sex acts, mean (SD)		6.7 (12.0)	11.7 (16.9)	12.0 (20.5)	F = 4.5	2,263	< .01
Number HIV-negative and status-unknown partners, mean (SD)		5.1 (9.5)	9.9 (12.0)	14.0 (16.9)	F = 11.1	2,304	< .001
Number of casual partners, mean (SD)		2.0 (2.8)	5.2 (9.4)	4.6 (8.4)	F = 5.3	2,317	< .01
Number of anonymous partners, mean (SD)		3.0 (9.6)	6.8 (10.7)	6.8 (12.7)	F = 4.1	2,311	< .05
**Psychosocial factors**							
Beck depression, mean (SD)		14.3 (9.4)	14.1 (9.4)	18.3 (10.7)	F = 6.0	2,298	< .01
Sexual compulsivity, mean (SD)		22.6 (7.9)	22.6 (6.6)	25.8 (7.5)	F = 4.5	2,198	< .01
Experiences of social stigma, mean (SD)		2.1 (.87)	2.1 (.83)	2.6 (.88)	F = 13.1	2,318	< .001

### Substance use variables

Men in the public-venues group reported using significantly more methamphetamine in a 30-day period and were more likely to have injected in the past two months than were men in the other two groups. The groups did not differ in binge use behavior or frequency of use. Men in the public-venues group were also significantly more likely to have used powder cocaine, crack cocaine, and heroin. A variety of other illicit drugs were used more frequently by men in the commercial-but-no-public-venues group. Specifically, men in this group were significantly more likely to report using poppers, special K, and GHB ("club drugs"). Finally, men in the private-venues-only group reported using significantly fewer illicit drugs than did men in the other two groups.

Men in the public-venues group reported consuming significantly more alcoholic drinks per day compared to the other two groups. Also, men in the public-venues group and men in the commercial-but-no-public-venues group were significantly more likely to report getting drunk during the previous two months as compared to men in the private-venues-only group. The three groups did not differ in frequency of alcohol consumption.

### Sexual risk behavior and partner types

Men in the public-venues group reported significantly more total unprotected sex. In addition, men in the public-venues group and men in the commercial-but-no-public-venues group reported significantly more unprotected anal sex and a greater number of HIV-negative and serostatus-unknown sexual partners as compared to their counterparts in the private-venues-only group. The three groups did not differ significantly in total number of unprotected oral or vaginal sex acts nor in number of sexually transmitted infections reported during the previous two months. Men in the commercial-venues and in the public-venues group had significantly larger numbers of casual and anonymous partners as compared to their counterparts in the private-venues-only group (see Table 1).

### Psychosocial factors

The public-venues group had significantly higher scores on depressive symptoms, sexual compulsivity, and experiences of social stigma. The "expectations of rejection" dimension of social stigma did not differ by sexual venue group.

## Discussion

The main finding of this research was the tendency for HIV-positive, methamphetamine-using MSM to have sexual encounters in more than one type of venue. Although a large majority of the men had had sex in a private venue, only one-third of the sample restricted itself to this type of venue. The majority of participants had had sex in private settings as well as in either a commercial or a public venue. This finding complements previous work that has described situations where MSM meet in commercial venues, such as bars or clubs, and later have sex in a public venue such as a park, beach, or alley [21]. Future studies should examine how sexual transactions evolve and how locations for sexual activity are decided upon. Individual characteristics and attitudes are likely to be pivotal; however, social, legal, and political environments are also likely to have a strong influence on individuals' decisions and actions [6].

About one-third of the sample had had at least one sexual encounter in a public place during the previous two months. This finding has implications for HIV-prevention efforts, given that MSM in the public-venues group had the highest levels of both sexual risk behavior and drug use behavior. Higher levels of sexual risk behavior in public venues may be associated with the limited opportunities available to outreach workers in public settings to provide HIV prevention messages and endorse safer sex norms, particularly when risk activities occur late at night [1,22]. One model of harm reduction for MSM that was tested in San Francisco involved late-night trips in a van to high-risk neighborhoods; workers distributed condoms and lubricant, exchanged needles, and provided harm-reduction information and HIV/STI testing to anyone who was out in public at that hour and was interested in receiving these items or services [23]. A similar outreach model could be tested in a number of types of public venue, including parks, public restroom areas, and beaches.

We noted that MSM who used public sex venues had higher levels of methamphetamine use, alcohol use and intoxication, and use of other illicit drugs, including crack cocaine and heroin. Previous research indicates that drunkenness and heavy drug use are not well tolerated in commercial settings such as bathhouses and sex clubs [24]. Methamphetamine-using MSM who arrive at commercial establishments severely intoxicated may be turned away or feel unwelcome there. Further, as did other researchers [12,25,26], we found that MSM in the commercial-but-no-public-venues group were more likely to use "club drugs." Although we did not ascertain whether these drugs were used in the venue, it is plausible to assume that stimulant drugs mix well with the general "party" atmosphere in sex clubs and bars.

Higher depression scores, greater experiences of social stigma, and higher sexual compulsivity scores reported by the public-venues group may be markers for internalized homophobia. Some of these men could be avoiding public, gay-oriented establishments in favor of venues where sex tends to be anonymous. Their greater perception of social stigma could reflect shame or discomfort about their sexual orientation [27]. Higher levels of sexual compulsivity reported by the same group is consistent with this hypothesis and with the findings of other researchers. Parsons and Halkitis [12] proposed that using public sex venues requires little planning and may be a convenient outlet for expressing compulsive behavior. If, as our data would suggest, the typical man who has unprotected sex with other men in public venues is depressed, not connected to the gay community, stigmatized, and highly compulsive, then condom-promotion efforts in gay-identified establishments are unlikely to reach this subgroup. The challenge for harm reduction interventions is to develop safer sex programs that promote individual behavior change while addressing stigma, discrimination, and mental health issues within the constraints of social and political environments.

Our findings indicate that the mean number of unprotected sex acts and unprotected anal sex acts in the past two months was highest among MSM who reported going to commercial but no public venues and among those who went to public venues. This should be interpreted with caution, because we did not ask participants whether they engaged in unprotected sex when "high" on methamphetamine in these venues. However, other studies have reported an association between having sex in a commercial venue and increased high-risk, unprotected sex [28,29].

Several studies have shown that commercial venues (e.g., bars, bathhouses) are appropriate settings for conducting HIV prevention education campaigns [12]. By contrast, conducting HIV prevention outreach in public settings is more controversial. Previous studies suggest that sexual behavior in public parks is highly secretive and occurs most often in secluded areas [30]. While these circumstances are challenging, a first step would be to implement discreet and appropriately placed condom vending machines and educational posters in public spaces that these men are known to frequent.

Our findings have several limitations. Although the sample was relatively large, our reliance on volunteers potentially limits generalizability. Data for sexual risk and drug use behaviors were self-reported, which might have resulted in underreporting. In the absence of prospective, longitudinal data, we cannot draw any conclusions regarding causal relationships between HIV risk behaviors and participants' use of sexual venues. Also, this research did not assess the extent to which participants had sex in these and other venues when not "high" on methamphetamine. Finally, this research used a two-month assessment window, which limits the reporting of activities. Some men might have been placed in a different sexual venue category if a longer recall period had been considered.

## Conclusions

These analyses enhance our understanding of the relationship between the use of different sexual venues and HIV risk behaviors in a sample of HIV-positive, MSM who had sex when they were "high" on methamphetamine. From a prevention perspective, ways must be found to promote safer sex in public venues. Structural interventions (such as condom machines) and social marketing campaigns are avenues that should be formally evaluated. Altering the "built environment" to discourage sex in public venues (e.g., removing doors in restrooms) may have the unintended consequence of driving men further underground and thus making them even less accessible to safer sex messages. Community interventions that promote acceptance of MSM lifestyles and address depressive symptoms and social stigma may be the best long-term option for reducing the need that some men feel to seek sex in public spaces. Further investigation should seek to enhance our understanding of the cultures that develop around venues where some HIV-positive men engage in sex in the context of methampethamine use. Research is also warranted into factors that are potentially modifiable (e.g., living arrangements, sexual compulsivity) to determine if attendance at risky sexual venues and concomitant HIV risk behaviors can be reduced by ameliorating these factors.

## Competing interests

The authors declare that they have no competing interests.

## Authors' contributions

SJS performed the analysis and wrote the first draft of the manuscript. SAS assisted in interpreting the analysis and in drafting the manuscript. JZ supervised data collection and assisted in drafting the manuscript. TP designed the intervention study, helped interpret the analysis results, and assisted in writing the manuscript. All authors read and approved the final manuscript.

## Pre-publication history

The pre-publication history for this paper can be accessed here:

http://www.biomedcentral.com/1471-2458/10/178/prepub
